# A systematic review of public health interventions to address breast cancer inequalities in low- and middle-income countries

**DOI:** 10.1186/s13643-024-02620-2

**Published:** 2024-07-25

**Authors:** Esther Z. Chanakira, Chloe V. Thomas, Julie Balen, Olena Mandrik

**Affiliations:** 1 School of Health and Related Research, University of Sheffield, 30 Regent St, Sheffield, S1 4DA UK; 2 School of Allied and Public Health Professions, Canterbury Christ Church University, Canterbury, UK; 3 Medical Research Council Unit The Gambia at the London, School of Hygiene and Tropical Medicine, Fajara, The Gambia; 4 Manmohan Memorial Institute of Health Sciences, Kathmandu, Nepal

**Keywords:** Breast cancer inequalities, Public health intervention, Low- and middle-income countries

## Abstract

**Background:**

Breast cancer is the most diagnosed cancer in the world, with a worse prognosis documented in low- and middle-income countries. Inequalities pertaining to breast cancer outcomes are observed at within-country level, with demographics and socioeconomic status as major drivers.

**Aim:**

This review aims to aggregate all available evidence from low- and middle-income countries on public health interventions that can be utilized to reduce breast cancer inequalities within the breast cancer continuum.

**Methods:**

The study was a systematic review and narrative synthesis of available literature, with the literature search conducted between September and October 2021. The search was re-run in September 2022 to update the review. PubMed, Scopus, Embase, African Index Medicus and LILACS were searched, based on predetermined criteria. Randomized controlled trials, cohort studies and quasi-experimental studies were included for review, while studies without an intervention and comparator group were excluded. The Joanna Briggs Institute family of checklists was used for quality assessment of the included studies. Data pertaining to study design, quality control and intervention effectiveness was extracted.

**Results:**

A total of 915 studies were identified for screening and 21 studies met the selection criteria. Only one study specifically evaluated the impact of an intervention on breast cancer inequalities. Diverse, multi-level interventions that can be utilized to address breast cancer inequalities through targeted application to disadvantaged subpopulations were identified. Educational interventions were found to be effective in improving screening rates, downstaging through early presentation as well as improving time to diagnosis. Interventions aimed at subsidizing or eliminating screening payments resulted in improved screening rates. Patient navigation was highlighted to be effective in improving outcomes throughout the breast cancer continuum.

**Conclusion:**

Findings from the systematic review underline the importance of early detection in breast cancer management for low- and middle-income countries. This can be achieved through a variety of interventions, including population education, and addressing access barriers to public health services such as screening, particularly among under-served populations. This study provides a comprehensive database of public health interventions relevant to low- and middle-income countries that can be utilized for planning and decision-making purposes. Findings from the review highlight an important research gap in primary studies on interventions aimed at reducing breast cancer inequalities in low- and middle-income countries.

**Systematic review registration:**

PROSPERO registration number: CRD42021289643.

**Supplementary Information:**

The online version contains supplementary material available at 10.1186/s13643-024-02620-2.

## Background

Breast cancer is the most diagnosed cancer and the leading cause of cancer deaths in women worldwide, with 2.3 million new cases recorded in 2020 and approximately 685,000 deaths [1]. In the same year, it was estimated to represent 11.4% of all global cancer cases and 6.9% of all global cancer deaths [1]. Inequalities in breast cancer outcomes occur both globally and within countries, with low- and middle-income countries (LMICs) experiencing worse outcomes compared to high-income countries (HICs). Inequalities are defined as avoidable differences in health outcomes between different population groups [2]. In LMICs, breast cancer is often characterized by the late presentation of disease and high mortality rates [2]. Only 20 to 50% of breast cancer patients are diagnosed at stages 1 and 2 in LMICs compared to an estimated 70% of patients diagnosed at these stages in HICs [3, 4]. Higher mortality rates in the overall population are observed in LMICs compared to HICs despite HICs having higher incidence rates [2]. The breast cancer age-standardized incidence rate is 88% higher in HICs compared to LMICs, whereas LMICs have a 17% higher age-standardized mortality rate in the overall population compared to HICs [1]. These observed differences can be explained through differences in exposure to risk factors and access to health care services, as well as contextual systemic factors impacting on women’s health [2, 5].

At the national level, inequalities in breast cancer outcomes are usually observed along socioeconomic and demographic lines for both LMICs and HICs [5]. These are present at all levels of the breast cancer continuum, starting from exposure to modifiable risk factors, all the way to access to palliative care and mortality rates [5]. Ultimately, women with a lower SES generally present with late-stage cancer and have a worse breast cancer prognosis compared to those with a higher SES [5]. A cohort study that followed breast cancer patients in sub-Saharan Africa documented inequalities in stage at diagnosis and access to treatment based on SES [6]. Differences in access to care relating to screening, early diagnosis, and treatment, due to affordability and access, are partially responsible for these observed inequalities [5, 6]. Women with a lower SES also tend to have more comorbidities, increasing their likelihood of dying from breast cancer, even when the cancer is diagnosed at the same stage as those with higher SES [7].

Individual-level cancer inequalities are generally driven by systemic social determinants of health, necessitating population-level public health interventions to address them. This review aims to aggregate and synthesize evidence from LMICs, on public health interventions which could reduce breast cancer inequalities pertaining to socioeconomic and demographic factors, within the breast cancer continuum. The World Bank country and lending groups (2021) were used to identify LMICs [8]. The review focuses on LMICs due to the documented poorer breast cancer prognosis (cancer fatality ratio) in those countries compared to HICs. The focus on inequalities was driven by the higher Gini Index coefficients observed in LMICs compared to HICs representing comparatively higher levels of inequalities. Healthcare systems in LMICs also tend to be oriented towards dealing with infectious diseases and have only recently started focusing on non-communicable diseases (NCDs), whereas HICs have been focused on NCDs for longer [2]. A review looking at healthcare delivery interventions to reduce cancer inequalities, specifically focusing on telemedicine and patient navigation, was identified in the literature [9]. While telemedicine and patient navigation have emerged as promising strategies to address health inequalities, there is a need for a broader array of interventions tailored to the specific contexts and needs for LMICs. In this review, we extend the scope beyond traditional approaches by encompassing a diverse range of public health interventions, including preventive, promotive and rehabilitative strategies, with a particular focus on their application and effectiveness in LMIC settings.

## Methods

The protocol for this systematic review and narrative synthesis was registered with the international prospective register of systematic reviews (PROSPERO, registration number CRD42021289643).

### Eligibility criteria

The population of interest for this review was adult women (over the age of 18) in LMICs. The review aimed to identify studies pertaining to any public health intervention that can be utilized to reduce socioeconomic and demographic inequalities in any breast cancer outcomes using appropriate comparator(s). Outcomes of interest included but were not limited to screening rates, time to diagnosis and treatment, stage at diagnosis and mortality. Public health interventions were defined as interventions aimed at improving health at population level, with both policy-based and research-based interventions included [10]. Population stratification as a methodological specification was not required for study inclusion due to the limited number of studies identified. The review was expanded beyond studies that were explicitly designed to address inequalities or those that demonstrated an improvement on subgroup inequalities due to the absence of studies within this scope. Studies that looked at interventions that can be applied selectively at the subgroup level without the study explicitly doing so were also included as they can be utilized to address inequalities.

Clinical studies aimed at determining the impact of a treatment intervention were excluded. Studies pertaining to the use of breast cancer self-examination and clinical breast examination as screening methods were also excluded due to a lack of sufficient evidence on the efficacy of breast self-examination and mixed results on the efficacy of clinical breast examination [11]. However, studies that included breast cancer self-examination and clinical breast examination as part of a broader public health intervention (e.g. an educational campaign) were included in the review. Randomized controlled trials (RCTs), cohort studies and quasi-experimental studies were reviewed, while case–control studies and cross-sectional survey studies were excluded. Papers appearing in peer-reviewed journals were included, and grey literature and conference abstracts were excluded. No restriction was placed on publication dates.

### Search strategy

The following electronic databases were searched between September and October 2021: PubMed, Scopus, Embase, African Index Medicus (AIM) and Latin American and Caribbean Health Sciences Literature (LILACS). The search was re-run in September 2022 to update the review. The InterTASC Information Specialists’ Sub-Group Search Filters as well as Medical Subject Heading (MeSH) terms relating to the Population Intervention Comparator Outcomes (PICO) question were used to formulate the PubMed search strategy. Appropriate elements of the place of residence, race/ethnicity/culture/language, occupation, gender or sex, religion, education, socioeconomic status, and social capital (PROGRESS-Plus) guidelines [12] were also included in formulating the search strategy. The Effective Practice and Organization of Care LMIC filters 2020 were used to limit identified studies to those conducted in LMICs [8]. The resulting search strategy was tested against a subset of identified papers that met the PICO requirements of the review. The PubMed search strategy was then adapted to meet the needs of the other electronic databases. Reference tracing was conducted using the reference lists of studies that met the inclusion criteria. Search strategies are included in Additional file 1.

### Study selection

Study selection was conducted in two phases, with the first phase consisting of abstract screening based on the exclusion criteria. The second phase was a review of the retrieved full texts based on the inclusion criteria. This was followed by extraction and synthesis for papers that met the selection criteria. Articles were screened using Rayyan, a systematic review software to improve efficiency [13], while references were managed using Mendeley. A liberal-accelerated approach to screening was used to double-check screening, with co-authors acting as second reviewers and checking the excluded full texts.

### Data extraction

Data extraction was carried out in Microsoft Excel using a standardized data extraction sheet which was developed to extract basic information pertaining to the study, as well as appropriate information on study methodology and outcomes. The effectiveness of the intervention was determined based on the author’s conclusion as well as whether the findings were statistically significant or not. Data extraction sheets are provided in Additional file 2.

### Quality assessment

The review included diverse study designs, intervention types and study outcomes. This made it difficult to identify a single appropriate quality assessment tool that could be used for the review. A family of checklists was used to allow for a balance between comparability across studies and appropriateness of the tool with regard to the study design. The Joanna Briggs Institute (JBI) family of checklists was used to assess the quality of studies identified during the review by the first reviewer [14].

### Data synthesis

All studies, regardless of quality assessment outcomes, were included in the narrative synthesis of the review due to the limited number of studies identified. Meta-analysis was not carried out for this review due to the heterogeneity of the interventions and study methods. Study outcomes are presented under the following categories: (i) studies pertaining to screening of asymptomatic individuals; (ii) studies pertaining to early detection of symptomatic patients; and (iii) studies pertaining to diagnostic and treatment adherence.

## Results

**Fig. 1:**
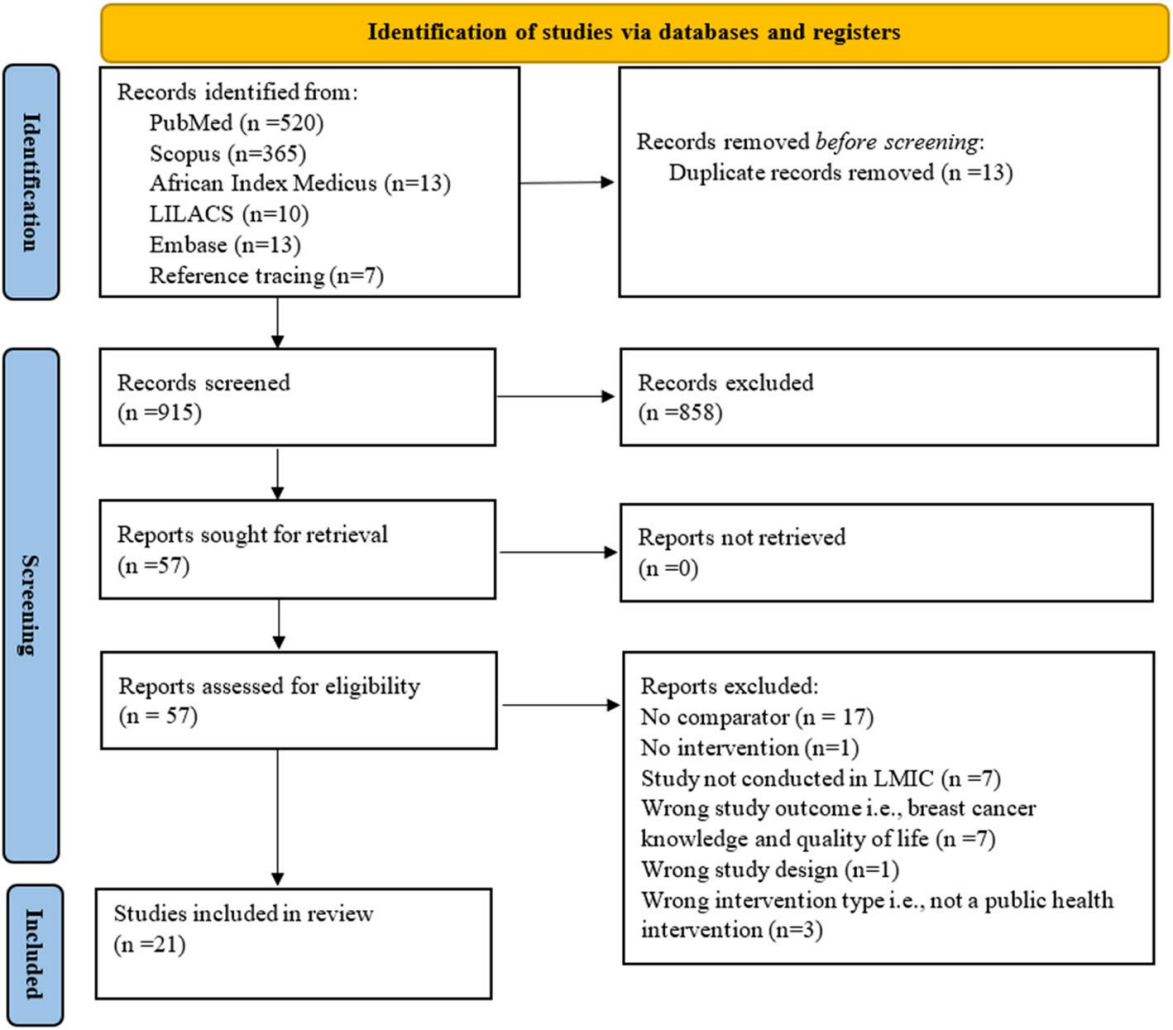
Prisma diagram depicting search and screening results and final study characteristics

A total of 915 records were identified for screening, and the final number of studies included in the review was 21, as illustrated in the PRISMA diagram depicting search and screening results and final study characteristics (Fig. 1).

### Study characteristics

Most of the studies identified were conducted in Asia (17/21 studies) with Iran (3 studies), and Turkey (4 studies) as the most common study sites. Only 3 studies were conducted in Africa (1 study in each of Rwanda, Sudan and Kenya), while 1 study was conducted in South America (Colombia).

Quasi-experimental studies and RCTs both represented 10 studies each from the 21 studies identified, with the remaining study being a cohort study comparing a prospective and retrospective cohort. Most of the studies identified (16 studies) targeted early detection through improving both screening rates and early symptomatic presentation. The rest of the studies (5 studies) focused on improving time to diagnosis, adherence to diagnostic procedure and adherence to treatment. Educational interventions focusing on improving knowledge, beliefs and behaviour were the most common intervention (13 studies) identified in the review. More than half of the studies (12 studies) identified interventions relating to screening uptake^1^ and attendance.^2^ One study looked at the impact of patient navigation on return for diagnosis, while the rest explored the impact of screening on stage at diagnosis.

### Screening of asymptomatic individuals

There was a total of 16 studies pertaining to the screening of asymptomatic individuals (Table 1). Only one study by Bao et al. was found to specifically address inequalities in screening attendance rates with a focus on the rural–urban axis of inequality [15]. It was a quasi-experimental study conducted in China that looked at the impact of an organized screening programme and cost-saving interventions in rural areas. The intervention resulted in an increase in likelihood of cancer screening, with women in the programme being 1.63 times more likely to receive screening compared to those who were not in the programme [15]. A significant reduction in inequalities in screening attendance between urban and rural women, with the relative inequality indicator decreasing by 40.8% and the absolute inequality indicator by 38.7%, was observed [15]. This was one of two studies that explored the most effective interventions in improving screening rates. The second study was a quasi-experimental study conducted in Jordan that looked at the impact of distributing free mammography vouchers after an educational intervention on screening [16]. Another intervention that was found to be highly effective in improving screening uptake was a multi-faceted work-based intervention offering motivational interviewing, printed educational material and navigation of patients within the healthcare system [17]. This intervention was evaluated using a quasi-experimental study conducted in China [17]. Compared to educational interventions that included an offer or invitation for screening, educational interventions without an explicit offer or invitation for screening were found to be relatively less effective in improving screening rates [18–23].

**Table 1:** Interventions pertaining to the screening of asymptomatic individuals

Study title	Intervention type	Outcome of interest	Results	Conclusion on effectiveness (compared to comparator)
Workplace-based breast cancer screening intervention in China [17]	Motivational interviewing, printed material and navigation	Mammography uptake	69% vs 4% (*p* < 0.001) ***statistically significant	Effective
Increasing breast cancer awareness and breast examination practices among women through health education and capacity building of primary healthcare providers: a pre-post intervention study in low socioeconomic area of Mumbai, India [18]	Educational intervention for both primary healthcare providers and women at risk of breast cancer	CBE attendance rate	19% pre-intervention vs 35.9% post-intervention	Effective
The effectiveness of a nurse-delivered breast health promotion program on breast cancer screening behaviours in non-adherent Turkish women: A randomized controlled trial [19]	Nurse-led educational health promotion programme	Screening attendance rate	Mammography screening: 15.5% intervention vs 9.7% control CBE: 11.3% intervention vs 6.5% control ***both not statistically significant	Effective (not statistically significant)
Effects of education based on the health belief model on screening behaviour in high-risk women for breast cancer, Tehran, Iran [20]	Educational session based on health belief model	Screening attendance rate	Mammography screening: 38% intervention vs 30% control ***not statistically significant CBE: 40% intervention vs 18% control ***statistically significant	Effective (not statistically significant)
Assessment of the effects of breast cancer training on women between the ages of 50 and 70 in Kemalpasa, Turkey [21]	Educational intervention for breast cancer and mammography training	Screening attendance rate	Mammography screening: 53.7% before intervention vs 58.5% after intervention CBE: 48.8% before intervention vs 48.8% after intervention ***both not statistically significant	Low effectiveness (not statistically significant)
Home visits to improve breast health knowledge and screening practices in a less privileged area in Jordan [16]	Culturally appropriate educational intervention and free mammography vouchers	Screening uptake	Mammography screening: 73% among those offered vouchers vs 2.66% among those not offered vouchers	Effective
A nationally quasi-experimental study to assess the impact of partial organized breast and cervical cancer screening programme on participation and inequalities [15]	Organized screening programme + cost-saving intervention in rural areas	Screening attendance rate (odds ratio)	Intervention increased screening attendance (OR = 1.63; 95%, 1.56–1.71) ***statistically significant % Change in Relative Index of Inequality: − 40.8% % Change in Slope Index of Inequality: − 38.7%	Effective
Interventional Education Methods for Increasing Women’s Participation in Breast Cancer Screening Program [24]	Individual education ± brochure for spouse vs group education	Mammography screening uptake	Mammography screening uptake rate: 20% individual education vs 22.3% individual education + brochure for spouse vs 33% for group education ***not statistically significant	Effective
Level of awareness of cervical and breast cancer risk factors and safe practices among college teachers of different states in India: do awareness programmes have an impact on adoption of safe practices? [22]	Educational intervention and CBE	Screening attendance rates	No change in CBE screening rates Mammography screening rate: 14% pre-intervention vs 22% post-intervention	Effective in increasing mammography screening rate
The Effect of Educational Intervention Based on the Theory of Planned Behaviour on Mammography Screening in Iranian Women [25]	Educational intervention	Mammography screening uptake	74% in intervention group vs 7% in control group (*p*-value < 0.05) ***statistically significant	Effective
The effect of two types of sms-texts on the uptake of screening mammogram: A randomized controlled trial [26]	General SMS-text inviting them to do a mammogram test only (intervention 1). General SMS-text inviting them to do a mammogram test + informative SMS-text informing participants about the benefits of mammogram screening and inviting them to do the mammogram test (intervention 2)	Mammography screening uptake	31.2% in intervention 1 group vs 30.7% in intervention 2 (*p*-value ≥ 0.05) ***not statistically significant Overall uptake rate from both groups–31.2%	Not effective
The effect of motivational interviewing on the change of breast cancer screening behaviours among rural Iranian women [23]	Two educational sessions and four weekly consecutive MI sessions on breast cancer screening in groups of 5–7 women	Screening attendance rate	0% control group vs 60% intervention group for CBE attendance rate 0% control group vs 26.7% intervention group for mammography attendance rate	Effective
Increased breast cancer screening and downstaging in Colombian women: A randomized trial of opportunistic breast-screening [27]	Opportunistic screening	Downstaging	Early breast cancer—2.1 (0.9–5.4) Advanced breast cancer—0.7 (0.2–2.4) All breast cancers—1.4 (0.7–2.8) ***non-statistically significant	Effective
An educational intervention based on the extended parallel process model to improve attitude, behavioural intention, and early breast cancer diagnosis: a randomized trial [28]	Educational intervention based on extended parallel process model	Downstaging	No significant improvement observed (*p*-value = 0.78)	Not effective
Cancer early detection program based on awareness and clinical breast examination: Interim results from an urban community in Mumbai, India [29]	Breast awareness brochures sent out annually + establishment of breast clinics in the PHC involved in the scheme	Downstaging	Advanced breast cancer—21.8% vs 20.8% ***statistically significant	Low effectiveness in downstaging
Effect of a breast navigation programme in a teaching hospital in Africa [30]	Patient navigation post-screening	Returning within 30 days Timely return (14 days) Mean time to return	Return within 30 days: OR, 4.43 (95%, 1.54–12.78) Statistically significant Timely return: OR, 2.85 (0.34–24.30) ***not statistically significant Mean time to return: 8.4 days vs 7.33 days ***not statistically significant	Effective in increasing return within 30 days

A quasi-experimental study conducted in Kenya found patient navigation to be effective in improving the proportion of patients returning for diagnosis within 30 days of screening [30]. In the same study, patients were 4.43 times as likely to return for diagnosis post-intervention compared to pre-intervention [24]. A non-statistically significant mean decrease in time to return of 1.07 days was also observed post-intervention [30].

Two interventions that were evaluated through cluster RCTs were found to be effective in downstaging. One of the studies which was conducted in Colombia explored the effectiveness of an opportunistic screening programme [30], while the other study which was conducted in Rwanda looked at a training programme for community health workers (CHWs), healthcare (HC) nurses and hospital clinicians [31]. Opportunistic screening resulted in a 25% reduction in advanced disease, compared to the control group, while the training programme aimed at healthcare workers resulted in a 27.4% reduction in advanced disease [27, 31]. The only intervention which was not effective in downstaging explored the impact of an educational intervention based on the extended parallel process model of social and behavioural theory on downstaging in Iran [28].

### Early diagnosis of symptomatic patients

Four cluster RCTs aiming to improve early diagnosis of symptomatic patients (3 from Asia and 1 from Africa) were identified (Table 2). All the interventions identified were effective in either increasing the symptomatic presentation rate or decreasing time to diagnosis post-symptomatic presentation. The interventions included were diverse, ranging from capacity building to self-education at patient level. The study by Pace et al. which was conducted in Rwanda found that giving breast cancer training to primary care workers resulted in downstaging in patients presenting with breast cancer. A cluster RCT study by Chowdhury et al. which explored the feasibility of case-finding in rural Bangladesh found that the use of motivational videos or the provision of patient navigation was feasible and resulted in a 41% increase in follow-up rates for diagnosis [32]. The study however did not separate the impact of patient navigation from that of the motivational videos. The cluster RCT by Ginsburg et al., which was also conducted in Bangladesh, found patient navigation in addition to cell-phone use by CHW to be effective in improving follow-up rates for diagnosis, with the intervention resulting in a 20% increase in follow-up rates [33]. A self-education intervention aimed at Indonesian women with breast cancer symptoms was also found to be effective in reducing the time to diagnosis, with a decrease of 13.26 days [34].

**Table 2:** Interventions aimed at early diagnosis of symptomatic patients and improving diagnostic and treatment adherence

Study title	Intervention type	Outcome of interest	Results	Conclusion on effectiveness (compared to comparator)
**Interventions aimed at early diagnosis of symptomatic patients**				
Cluster Randomized Trial to Facilitate Breast Cancer Early Diagnosis in a Rural District of Rwanda [31]	Training for CHWs, HC nurses and hospital clinicians	Patient volume and downstaging	1,486 unique patients visited intervention HCs for breast concerns (537.1 patients/100,000 person-years) vs 315 patients (104.0 patients/100,000 person-years) who visited control HCs (*p* < 0.001) 47.4% stage 1 and 2 disease in intervention group vs 20% stage 1 and 2 disease in control group	Effective
Feasibility Study of Case-Finding for Breast Cancer by Community Health Workers in Rural Bangladesh [32]	Cell-phone programme + / − motivational video OR navigation	Follow-up rates for diagnosis	Follow-up rates: 25% in control group, 66% in cell-phone programme + motivational video OR navigation group	Effective
A self-help intervention for reducing time to diagnosis in Indonesian women with breast cancer symptoms [34]	Self-health educational intervention	Time to diagnosis	Difference in time to diagnosis between intervention and control group: − 13.26 days (95% CI, − 24.51 to − 2.00)	Effective
An mHealth model to increase clinic attendance for breast symptoms in rural Bangladesh: can bridging the digital divide help close the cancer divide? [33]	Arm A: CHW with smart phone with applications to guide interview, report data, show motivational video, and offer appointment for women with an abnormal CBE Arm B: smart phone/applications identical to Arm A plus CHW had training in “patient navigation” to address potential barriers to seeking care	Follow-up rates for diagnosis	Arm A—107 (43%) Arm B—152 (63%) Arm C (control)—37 (53%) *P*-value for A vs B is *p* < 0.0001 ***statistically significant	Effective
**Interventions aimed at improving diagnostic and treatment adherence**				
Feasibility of Patient Navigation to Improve Breast Cancer Care in Malaysia [35]	Patient navigation post-screening	Proportion meeting performance indicators for diagnosis Defaulting treatment	Proportion meeting performance indicators for mammography: 96.4% current cohort vs 74.4% historical cohort (*p*-value < 0.001) Proportion meeting performance indicators for biopsy: 92.5% current cohort vs 76.1% historical cohort (*p*-value < 0.003) Proportion of patients defaulting on treatment: 4.4% current cohort vs 11.5% historical cohort (*p*-value = 0.048)	Effective

### Interventions aimed at improving diagnostic and treatment adherence

Only one study in the form of a cohort study conducted in Malaysia was found in this category (Table 2). The study looked at the impact of patient navigation on diagnostic and treatment adherence. The proportion of patients meeting performance indicators relating to mammography and biopsy increased by 25.5% and 16.4%, respectively [35]. The proportion of patients defaulting on treatment also decreased by 7.1% [35].

### Study quality

**Fig. 2:**
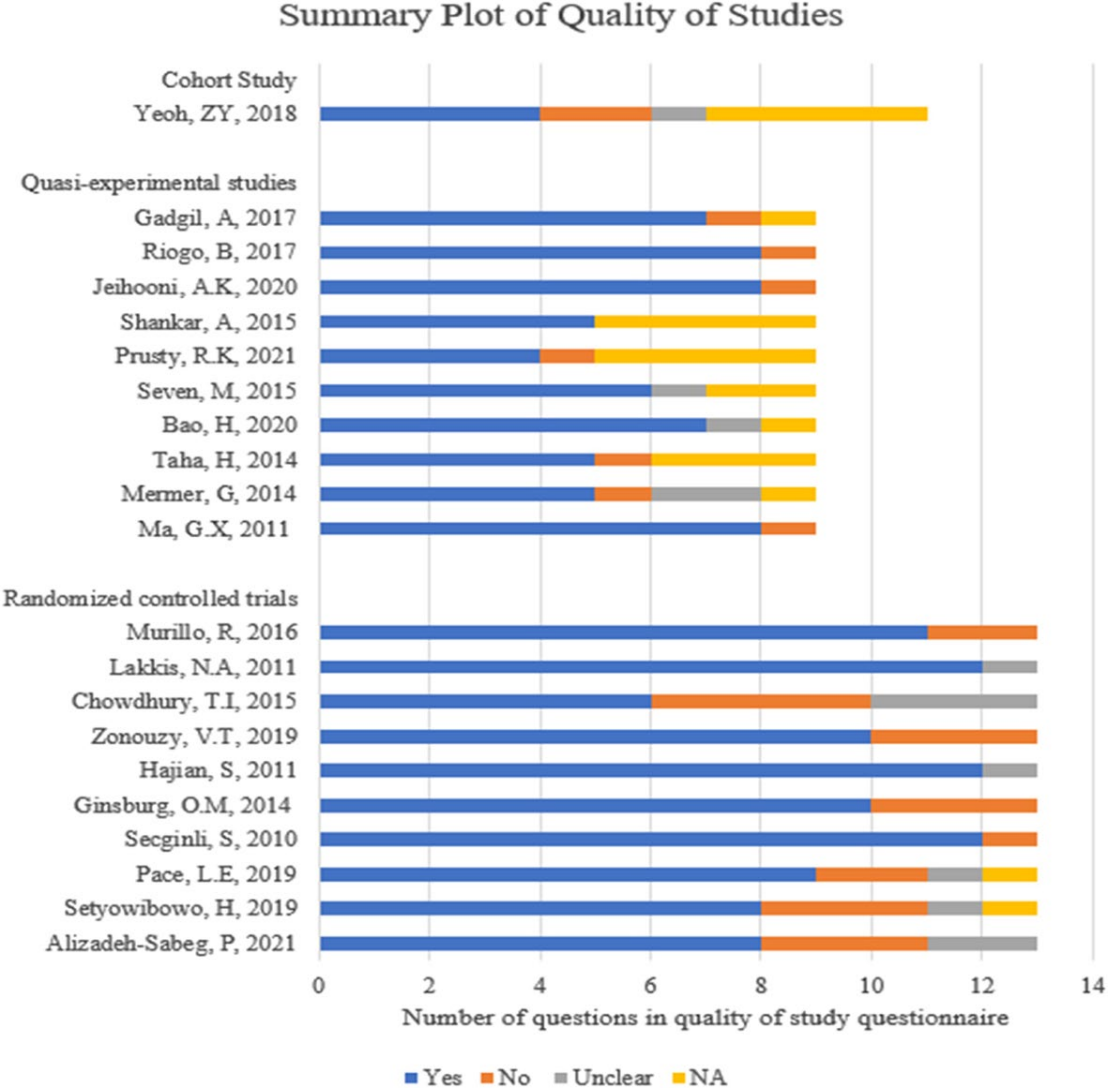
Summary plot of quality of studies

The RCTs identified in the review were of mixed quality with criteria relating to the blinding of participants, outcome assessors and individuals delivering the intervention not being commonly met in the studies. This was mostly because educational interventions, which limit the extent of blinding, were relatively common in the studies included in this review. Three studies [23, 32, 34] were unclear on randomization and allocation concealment to the study groups. The cohort study [35] was of low quality; it did not provide baseline characteristics of the historical cohort to allow for comparison with the prospective cohort. Confounding factors were also not explored in the study. The quasi-experimental studies [15–18, 21, 22, 24, 25, 29, 30] were of mixed quality, with some of the studies identified using the same group of participants with measurements taken before and after the intervention [16, 18, 21, 22, 29, 30]. Tables depicting study quality are available in Additional file 3. Figure 2 shows a summary plot of the quality of studies identified in the review.

## Discussion

This review aimed to identify public health interventions within the breast cancer continuum that could be utilized to reduce breast cancer inequalities in a LMIC context. Most of the studies included in the review focused on improving screening rates and early diagnosis with only one study focusing on improving treatment adherence. Findings from this systematic review suggest that educational interventions are effective in improving screening rates (both screening attendance and screening uptake rates), and downstaging through early presentation and improved time to diagnosis. Educational interventions without an explicit offer or invitation for screening were found to be less effective compared to those that had an explicit offer or invitation for screening, highlighting the presence of barriers in access to care beyond knowledge and awareness, for example, accessibility or affordability. The observed effectiveness of educational interventions in improving screening rates is supported by findings from a scoping review conducted in the USA which looked at interventions aimed at improving screening in rural communities; that study also found educational interventions to be effective [36]. Seven et al. found that an educational intervention at the group level is more effective in improving mammography uptake compared to interventions provided at the individual level [24]. This is an especially important finding in resource-constrained settings, as group level interventions that require less resources can be provided instead of more costly individual-level interventions.

The identified cluster RCTs which were of medium to high quality demonstrated a statistically significant positive impact of smartphone applications and training programmes for medical personnel on the follow-up rate and time to diagnosis for symptomatic patients. Though only a few identified studies explored these surrogate outcomes, the shorter time to diagnosis observed is likely to lead to downstaging as well as decreased breast cancer mortality. For example, Unger-Saldana et al. found that delays within the diagnostic pathway were associated with advanced disease at diagnosis in Mexico [37], while a systematic review by Richards et al. concluded that longer delays within the diagnosis and treatment pathway were associated with lower survival [38].

This systematic review has demonstrated a lack of available evidence on the impact of public health interventions on inequalities in LMICs. Only one identified study by Bao et al. specifically looked at the impact of an intervention on reducing inequalities between subgroups [15]. This study concluded that organized screening with a subsidized cost is effective in decreasing the magnitude of inequalities between rural and urban women through addressing affordability and physical barriers in accessing care. This is supported by findings from another systematic review by Bygrave et al. that looked at the impact of interventions on inequalities in cancer-related outcomes in HICs and found that organized screening services resulted in a decrease in inequalities [39].

Importantly, this systematic review illustrates that interventions that have a positive impact on breast cancer outcomes at the population or group level can have a negative impact on inequalities. Murillo et al. found opportunistic screening to be effective in downstaging; however, opportunistic screening is associated with an increase in socioeconomic inequalities due to the high correlation between education and income [40]. Screening services are often associated with an increase in socioeconomic inequalities in cancer, with a study conducted in the UK finding that inequalities pertaining to colorectal cancer increased as screening effectiveness improved [41]. To reduce inequality, high levels of coverage are required which is resource intensive [40]. This phenomenon is supported by the inverse equity hypothesis which states that new health interventions are initially accessed by the rich before trickling down to the poor, with inequalities narrowing as the interventions diffuse to the poor [40].

Educational interventions were the most common intervention aimed at improving screening uptake and attendance, with only one study identified that did not include an educational component [15]. Diverse approaches were taken in designing the interventions with some focusing on social and behavioural theories, while others explored education at the group level versus the individual level [16, 17, 20, 25]. The study by Taha et al. was the only one to specify that the educational intervention provided was culturally tailored [16]. Yet, this is an important factor when considering the effectiveness of the intervention within the study setting as well as transferability of findings from the studies given the cultural diversity of LMICs [42]. Studies with interventions that aimed to improve early diagnosis in symptomatic patients used surrogate outcomes such as patient volume and changes in follow-up rates. This requires an inference on the relationship between these surrogate outcomes and cancer outcomes such as stage at diagnosis and mortality rates. This relationship is not always guaranteed to be positive due to contextual factors such as health system capacity.

Having only identified one study by Bao et al. that focused on the impact of a public health intervention on breast cancer inequalities in this review, the inclusion of further evidence collected from HICs pertaining to breast cancer inequalities would have allowed for comparison of interventions that are effective in LMICs and those that are effective in HICs, providing further understanding of the relationship between study context and interventions. A strength of the study was the use of multiple databases for the literature search, including regional indices such as LILACs, limiting the likelihood of missing relevant studies. A methodological limitation of this review centres on the diverse nature of the interventions and outcomes considered, which did not allow for meta-analysis to be performed and limited the extent to which the studies could be integrated and compared in the narrative review. However, this is also a strength as the review provides a comprehensive overview of different types of interventions.

## Conclusion

From the identified studies, educational interventions were found to be effective in improving both screening attendance and screening uptake. The review highlights the role played by public health interventions in breast cancer management and inequalities by extension in LMICs. Studies identified in this review can be used to inform policy pertaining to breast cancer inequalities as well as forming a basis for further research in the form of pilot studies or economic evaluations in similar research settings. Further research on interventions specifically aimed at addressing breast cancer inequalities in LMICs should be conducted as only one study was identified that specifically explored inequality. Overall, the findings from the systematic review highlight the importance of early detection in breast cancer management for low- and middle-income countries.

### Supplementary Information

Additional file 1. Search strategies.

Additional file 2. Data extraction sheets.

Additional file 3. JBI quality assessment.

## Data Availability

Data sharing is not applicable to this article as no datasets were generated or analyzed during the current study.

## Abbreviations

LMIC: Low- and middle-income countries
HIC: High-income countries
SES: Socioeconomic status
CHW: Community health workers
CBE: Clinical breast examination
RCT: Randomized controlled trial
AIM: African Index Medicus
LILACS: Latin American and Caribbean Health Sciences Literature
PICO: Population Intervention Comparator Outcome(s)

## Footnotes

1 Screening/Mammography/CBE Uptake refers to percentage of women who have been offered/invited for screening and have attended in response to the offer/invite.

2 Screening/Mammography/CBE Attendance Rate refers to the percentage of women who attended screening after an intervention which improved knowledge but did not explicitly offer/invite participants for screening.
