# Supplementary material for: A systematic review of public health interventions to address breast cancer inequalities in low- and middle-income countries
Source: Syst Rev. 2024 Jul 25;13:195. doi: 10.1186/s13643-024-02620-2 (PMC11271015; doi:10.1186/s13643-024-02620-2)
Supplement: Supplementary file 2 — Additional file 2. Data extraction sheets. [file 13643_2024_2620_MOESM2_ESM.docx]

Table 1: Interventions pertaining to population screening.

| **Study title** | **First Author, Year and Ref** | **Study setting** | **Study type** | **Intervention type** | **Study period** | **Outcome of interest** | **Results** | **Conclusion on effectiveness** |
| --- | --- | --- | --- | --- | --- | --- | --- | --- |
| Workplace-based breast cancer screening intervention in China | Ma, G.X, 2011 | China (n=453) | Quasi-experimental study | Motivational interviewing, printed material, and navigation | 6 months | Mammography uptake | 69% vs 4% (p<0.001)  ***statistically significant. | Effective |
| Increasing breast cancer awareness and breast examination practices among women through health education and capacity building of primary healthcare providers: a pre-post intervention study in low socioeconomic area of Mumbai, India | Prusty, R.K, 2021 | India (n=480) | Quasi-experimental study | Educational intervention for both primary healthcare providers and women at risk of breast cancer. | 6 months | CBE attendance rate | 19% pre-intervention vs 35.9% post-intervention. | Effective |
| The effectiveness of a nurse-delivered breast health promotion program on breast cancer screening behaviours in non-adherent Turkish women: A randomized controlled trial | Secginli, S, 2010 | Turkey (n=190) | Randomized controlled trial | Nurse-led educational health promotion program | 6 months | Screening attendance rate | Mammography screening: 15.5% intervention vs 9.7% control.  CBE: 11.3% intervention vs 6.5% control.  *** both not statistically significant. | Effective (not statistically significant) |
| Effects of education based on the health belief model on screening behaviour in high-risk women for breast cancer, Tehran, Iran | Hajian, S, 2011 | Iran (n=100) | Randomized controlled trial | Educational session based on Health Belief Model | 3 months | Screening attendance rate | Mammography screening: 38% intervention vs 30% control.  *** not statistically significant.  CBE: 40% intervention vs 18% control.  ***statistically significant. | Effective (not statistically significant) |
| Assessment of the effects of breast cancer training on women between the ages of 50 and 70 in Kemalpasa, Turkey. | Mermer, G, 2014 | Turkey (n=106) | Quasi-experimental study | Educational intervention for breast cancer and mammography training | ≈1 year, 11 months | Screening attendance rate | Mammography screening: 53.7% before intervention vs 58.5% after intervention.  CBE: 48.8% before intervention vs 48.8% after intervention.  *** both not statistically significant. | Low effectiveness (not statistically significant) |
| Home visits to improve breast health knowledge and screening practices in a less privileged area in Jordan. | Taha, H, 2014 | Jordan (n=2400) | Quasi-experimental study | Culturally appropriate educational intervention and free mammography vouchers | 6 months | Screening uptake | Mammography screening: 73% among those offered vouchers vs 2.66% among those not offered vouchers. | Effective |
| A nationally quasi-experimental study to assess the impact of partial organized breast and cervical cancer screening programme on participation and inequalities. | Bao, H. 2020 | China (n=69,875) | Quasi-experimental study | Organized screening program + cost-saving intervention in rural areas | 6 years | Screening attendance rate (odds ratio) | Intervention increased screening attendance (OR=1.63, 95%: 1.56-1.71)  ***statistically significant  % Change in Relative Index of Inequality: - 40.8%  % Change in Slope Index of Inequality: -38.7% | Effective |
| Interventional Education Methods for Increasing Women's Participation in Breast Cancer Screening Program. | Seven, M, 2015 | Turkey (n=327) | Quasi-experimental study | Individual education +/- brochure for spouse vs group education | 3 months | Mammography screening uptake | Mammography screening uptake rate: 20% individual education vs 22.3% individual education + brochure for spouse vs 33% for group education.  ***not statistically significant | Effective (not statistically significant) |
| Level of awareness of cervical and breast cancer risk factors and safe practices among college teachers of different states in India: do awareness programmes have an impact on adoption of safe practices? | Shankar, A, 2015 | India (n=156) | Quasi-experimental study | Educational intervention and CBE | 1 year | Screening attendance rates | No change in CBE screening rates.  Mammography screening rate: 14% pre-intervention vs 22% post intervention. | Effective in increasing mammography screening rate. |
| The Effect of Educational Intervention Based on the Theory of Planned Behaviour on Mammography Screening in Iranian Women | Jeihooni,A.K., 2020 | Iran (n=400) | Quasi-experimental study | Educational intervention | 6 months | Mammography screening uptake | 74% in intervention group vs 7% in control group (p-value<0.05).  ***Statistically significant. | Effective |
| The effect of two types of sms-texts on the uptake of screening mammogram: A randomized controlled trial | Lakkis, N.A. 2011 | Lebanon (n=385) | Randomized controlled trial | General SMS-text inviting them to do a mammogram test only (Intervention 1)  General SMS-text inviting them to do a mammogram test + informative SMS-text informing participants about the benefits of mammogram screening and inviting them to do the mammogram test (intervention 2) | Intervention delivered over 3 months with a follow up of 6 months | Mammography screening uptake | 31.2% in Intervention 1 group vs 30.7% in Intervention 2 (p-value≥0.05).  ***not statistically significant  Overall uptake rate from both groups - 31.2%. | Not effective (not statistically significant) |
| The effect of motivational interviewing on the change of breast cancer screening behaviours among rural Iranian women | Alizadeh-Sabeg, P. 2021 | Iran (n=120) | Cluster randomized controlled trial | Two educational sessions and four weekly consecutive MI sessions on breast cancer screening in groups of 5–7 women. | Intervention period - 3 months. 8 weeks between intervention and post-intervention survey. | Screening attendance rate | 0% control group vs 60% intervention group for CBE attendance rate.  0% control group vs 26.7% intervention group for mammography attendance rate. | Effective |
| Increased breast cancer screening and downstaging in Colombian women: A randomized trial of opportunistic breast-screening | Murillo,R. 2016 | Columbia (n=7,436) | Cluster randomized controlled trial | Organized opportunistic screening | 4 years | Downstaging | Early breast cancer - 2.1(0.9-5.4).  Advanced breast cancer - 0.7(0.2-2.4)  All breast cancers - 1.4(0.7-2.8)  ***non-statistically significant | Effective but not statistically significant |
| An educational intervention based on the extended parallel process model to improve attitude, behavioural intention, and early breast cancer diagnosis: a randomized trial | Zonouzy, V.T, 2019 | Iran (n=438) | Cluster randomised controlled trial | Educational intervention based on extended parallel process model | 3 months | Downstaging | No significant improvement observed (p-value =0.78) | Not effective |
| Cancer early detection program based on awareness and clinical breast examination: Interim results from an urban community in Mumbai, India | Gadgil, A. 2017 | India (n=22,500) | Quasi-experimental study (before and after study) | Breast awareness brochures sent out annually + establishment of breast clinics in the PHC involved in the scheme | 3 years | Downstaging | Advanced breast cancer – 21.8% vs 20.8%  ***statistically significant | Low effectiveness in downstaging |
| Effect of a breast navigation programme in a teaching hospital in Africa | Riogi, B, 2017 | Kenya (n=76) | Quasi-experimental study | Patient navigation post-screening | 6 months | Returning within 30 days.  Timely return (14 days).  Mean time to return. | Return within 30 days: OR: 4.43 (95%: 1.54-12.78)  Statistically significant.  Timely return: OR: 2.85(0.34-24.30).  *** not statistically significant.  Mean time to return: 8.4 days vs 7.33 days.  *** not statistically significant. | Effective in increasing return within 30 days. |

Table 2: Interventions aimed at early diagnosis of symptomatic patients

| **Study title** | **First Author, Year and Ref** | **Study setting (n = study participants OR clusters were appropriate)** | **Study type** | **Intervention type** | **Study period** | **Outcome of interest** | **Results** | **Conclusion on effectiveness** |
| --- | --- | --- | --- | --- | --- | --- | --- | --- |
| Cluster Randomized Trial to Facilitate Breast Cancer Early Diagnosis in a Rural District of Rwanda | Pace, L.E, 2019, (14) | Rwanda  (n=18 healthcare centres) | Cluster randomized trial | Training for CHWs, HC nurses and hospital clinicians | 2 years | Patient volume and Downstaging | 1,486 unique patients visited intervention HCs for breast concerns (537.1 patients/100,000 person-years) v 315 patients (104.0 patients/100,000 person years) who visited control HCs (P < 0.001).  47.4% stage 1 and 2 disease in intervention group vs 20% stage 1 and 2 disease in control group. | Effective |
| Feasibility Study of Case-Finding for Breast Cancer by Community Health Workers in Rural Bangladesh | Chowdhury, T.I, 2015, (19) | Bangladesh (n=3,150) | Cluster randomized trial | Cell-phone program +/- motivational video OR navigation | Not stated | Follow up rates for diagnosis | Follow up rates: 25% in control group, 66% in cell-phone program + motivational video OR navigation group. | Effective |
| A self-help intervention for reducing time to diagnosis in Indonesian women with breast cancer symptoms | Setyowibowo, H, 2019, (18) | Indonesia (n=132) | Cluster randomized trial (cross-over study) | Self-health educational intervention | 5 months and 8 months | Time to diagnosis | Difference in time to diagnosis between intervention and control group: -13.26 days (95%CI: -24.51 to -2.00). | Effective |
| An mHealth model to increase clinic attendance for breast symptoms in rural Bangladesh: can bridging the digital divide help close the cancer divide? | Ginsburg, O.M, 2014, (37) | Bangladesh (n=22,337) | Cluster randomized controlled trial | **Arm A**: CHW with smart phone with applications to guide interview, report data, show motivational video, and offer appointment for women with an abnormal CBE.  **Arm B**: smart phone/applications identical to Arm A plus CHW had training in “patient navigation” to address potential barriers to seeking care. | 4 months | Follow up rates for diagnosis | Arm A - 107(43%)  Arm B - 152(63%)  Arm C (control)- 37(53%)  p-value for A vs B is p<0.0001.  ***statistically significant | Effective |

Table 3: Treatment and diagnostics adherence

| **Study title** | **First Author, Year and Ref** | **Study setting** | **Study type** | **Intervention type** | **Study period** | **Outcome of interest** | **Results** | **Conclusion on effectiveness** |
| --- | --- | --- | --- | --- | --- | --- | --- | --- |
| Feasibility of Patient Navigation to Improve Breast Cancer Care in Malaysia | Yeoh, Z, 2018, (24) | Malaysia (n=238) | Cohort study | Patient navigation post-screening | 2 years (1 year for intervention and 1 year for retrospective comparative cohort) | Proportion meeting performance indicators for diagnosis.  Defaulting treatment. | Proportion meeting performance indicators for mammography: 96.4% current cohort vs 74.4% historical cohort (p-value < 0.001).  Proportion meeting performance indicators for biopsy: 92.5% current cohort vs 76.1% historical cohort (p-value < 0.003).  Proportion of patients defaulting on treatment: 4.4% current cohort vs 11.5% historical cohort (p-value=0.048). | Effective |
