# Supplementary material for: A systematic review of public health interventions to address breast cancer inequalities in low- and middle-income countries
Source: Syst Rev. 2024 Jul 25;13:195. doi: 10.1186/s13643-024-02620-2 (PMC11271015; doi:10.1186/s13643-024-02620-2)
Supplement: Supplementary file 3 — Additional file 3. JBI quality assessment. [file 13643_2024_2620_MOESM3_ESM.docx]

*Table 1:* *Study quality of Randomized Controlled Trials (JBI criteria)^^[[1]](#footnote-1)^^*

| **First Author** | **True RA** | **Concealed allocation to TG** | **Similarity between TGs at baseline** | **Blinding of participants** | **Blinding of those delivering treatment** | **Blinding of OAs** | **Identical Treatment of Groups except intervention** | **Complete follow-up** | **Analysis of participants in groups to which they were randomly assigned** | **Same measurement for TGs** | **Outcomes measured in reliable way** | **Appropriate statistics** | **Appropriate trial design** | **Overall rating** |
| --- | --- | --- | --- | --- | --- | --- | --- | --- | --- | --- | --- | --- | --- | --- |
| Alizadeh-Sabeg, P, 2021 | ? | ? | Y | N | N | N | Y | Y | Y | Y | Y | Y | Y | 8Y |
| Setyowibowo, H, 2019 | ? | NA | Y | N | N | N | Y | Y | Y | Y | Y | Y | Y | 8Y |
| Pace, L.E, 2019 | Y | N | Y | NA | N | ? | Y | Y | Y | Y | Y | Y | Y | 9Y |
| Secginli, S, 2010 | Y | N | Y | Y | Y | Y | Y | Y | Y | Y | Y | Y | Y | 12Y |
| Ginsburg, O.M, 2014 | Y | Y | Y | N | N | Y | Y | Y | Y | Y | Y | N | Y | 10Y |
| Hajian, S, 2011 | Y | Y | ? | Y | Y | Y | Y | Y | Y | Y | Y | Y | Y | 12Y |
| Zonouzy, V.T, 2019 | Y | Y | Y | N | N | N | Y | Y | Y | Y | Y | Y | Y | 10Y |
| Chowdhury, T.I, 2015 | ? | ? | N | ? | N | N | Y | N | Y | Y | Y | Y | Y | 6Y |
| Lakkis, N.A, 2011 | Y | Y | Y | Y | Y | ? | Y | Y | Y | Y | Y | Y | Y | 12Y |
| Murillo, R, 2016 | Y | N | Y | Y | N | Y | Y | Y | Y | Y | Y | Y | Y | 11Y |

*Table 2: Study quality of Quasi-experimental studies (JBI criteria)*

| **First Author** | **Clear cause and effect** | **Similar participants included in comparison** | **Similar treatment of groups except for intervention** | **Control group present** | **Multiple measurements of outcome** | **Complete follow-up and adequate description of differences** | **Outcomes of participants included in any comparison measured in same way** | **Outcomes measured in reliable way** | **Appropriate statistics** | **Overall rate** |
| --- | --- | --- | --- | --- | --- | --- | --- | --- | --- | --- |
| Ma, G.X, 2011 | Y | Y | Y | Y | N | Y | Y | Y | Y | 8Y |
| Mermer, G, 2014 | Y | ? | Y | NA | N | ? | Y | Y | Y | 5Y |
| Taha, H, 2014 | Y | NA | NA | NA | N | Y | Y | Y | Y | 5Y |
| Bao, H, 2020 | Y | ? | Y | Y | NA | Y | Y | Y | Y | 7Y |
| Seven, M, 2015 | Y | ? | Y | Y | NA | Y | NA | Y | Y | 6Y |
| Prusty, R.K, 2021 | Y | NA | NA | NA | N | Y | NA | Y | Y | 4Y |
| Shankar, A, 2015 | Y | NA | NA | NA | Y | Y | NA | Y | Y | 5Y |
| Jeihooni, A.K, 2020 | Y | Y | Y | Y | N | Y | Y | Y | Y | 8Y |
| Riogo, B, 2017 | Y | Y | Y | Y | N | Y | Y | Y | Y | 8Y |
| Gadgil, A, 2017 | Y | Y | Y | NA | N | Y | Y | Y | Y | 7Y |

*Table 3:* *Study quality of Cohort Studies (JBI criteria)*

| **First Author** | **Similarity between TGs at baseline** | **Exposures measured similarly between groups** | **Exposure measured in valid and reliable way** | **Confounders identified** | **Strategies to deal with confounders stated** | **Participants free of outcome at start of study** | **Outcomes measured in a valid and reliable way** | **Follow up time reported and sufficiently long** | **Complete follow up** | **Strategies to address incomplete follow up** | **Appropriate statistics** | **Overall rating** |
| --- | --- | --- | --- | --- | --- | --- | --- | --- | --- | --- | --- | --- |
| Yeoh, Z. (1) | ? | NA | Y | N | N | NA | Y | Y | NA | NA | Y | 4Y |

1. Abbreviations: Y-Yes, N-No, ?-Unclear, NA-Not applicable, OAs-Outcome assessors, RA-Random assignment, TG- Treatment group [↑](#footnote-ref-1)
